# Ivy gourd (*Coccinia grandis* L. Voigt) root suppresses adipocyte differentiation in 3T3-L1 cells

**DOI:** 10.1186/1476-511X-13-88

**Published:** 2014-05-28

**Authors:** Ruthaiwan Bunkrongcheap, Nongporn Hutadilok-Towatana, Kusumarn Noipha, Chatchai Wattanapiromsakul, Masashi Inafuku, Hirosuke Oku

**Affiliations:** 1 Department of Biochemistry, Faculty of Science, Prince of Songkla University, Hat-Yai 90110, Thailand; 2 Natural Product Research Center of Excellence, Prince of Songkla University, Hat-Yai 90110, Thailand; 3 Faculty of Health and Sports Science, Thaksin University, Patthalung 93110, Thailand; 4 Department of Pharmacognosy and Pharmaceutical Botany, Faculty of Pharmaceutical Sciences, Prince of Songkla University, Hat-Yai 90110, Thailand; 5 Center of Molecular Biosciences, Tropical Biosphere Research Center, University of the Ryukyus, Okinawa 903-0213, Japan

**Keywords:** Anti-adipogenesis, Adipocyte, Ivy gourd, Obesity, 3T3-L1 cells

## Abstract

**Background:**

Ivy gourd (*Coccinia grandis* L. Voigt) is a tropical plant widely distributed throughout Asia, Africa, and the Pacific Islands. The anti-obesity property of this plant has been claimed but still remains to be scientifically proven. We therefore investigated the effects of ivy gourd leaf, stem, and root on adipocyte differentiation by employing cell culture model.

**Methods:**

Dried roots, stems, and leaves of ivy gourd were separately extracted with ethanol. Each extract was then applied to 3T3-L1 pre-adipocytes upon induction with a mixture of insulin, 3-isobutyl-1-methylxanthine, and dexamethasone, for anti-adipogenesis assay. The active extract was further fractionated by a sequential solvent partitioning method, and the resulting fractions were examined for their abilities to inhibit adipogenesis in 3T3-L1 cells. Differences in the expression of adipogenesis-related genes between the treated and untreated cells were determined from their mRNA and protein levels.

**Results:**

Of the three ivy gourd extracts, the root extract exhibited an anti-adipogenic effect. It significantly reduced intracellular fat accumulation during the early stages of adipocyte differentiation. Together with the suppression of differentiation, expression of the genes encoding PPARγ, C/EBPα, adiponectin, and GLUT4 were down-regulated. Hexane-soluble fraction of the root extract also inhibited adipocyte differentiation and decreased the mRNA levels of various adipogenic genes in the differentiating cells.

**Conclusions:**

This is the first study to demonstrate that ivy gourd root may prevent obesity based mainly on the ability of its active constituent(s) to suppress adipocyte differentiation *in vitro*. Such an inhibitory effect is mediated by at least down-regulating the expression of PPARγ-the key transcription factor of adipogenesis in pre-adipocytes during their early differentiation processes.

## Background

Obesity, an abnormal excessive increase of adipose tissue, is an important risk factor that contributes to the development of atherosclerosis, fatty liver, hyperlipidemia, diabetes mellitus, hypertension, inflammation, and various types of cancer [1]. It is characterized at the cellular level by an increase in the number and/or size of adipocytes, round lipid-filled cells, that differentiate from their fibroblast-like precursor cells present in adipose tissue. Therefore, reducing the differentiation into adipocytes or anti-adipogenesis and/or increasing the intracellular lipid breakdown or adipolysis are possible anti-obesity mechanisms. Despite the fact that anti-obesity medication is an effective therapeutic approach, most prescribed drugs have adverse side effects. These limitations have consequently motivated investigations into the search for ingredients from natural sources that can regulate adipocyte function especially those that can suppress adipogenesis [2-8].

Ivy gourd (*Coccinia grandis* L. Voigt or *C. cordifolia* L. Cogn. or *C. indica* Wight & Arn. or *Cephalandra indica* Naud.) is a perennial plant in the family Cucurbitaceae, abundantly present in tropical countries likes India, Indonesia, Malaysia, the Philippines, and Thailand. It is a climbing vine with tuberous roots, and fruits throughout the year. The South-East Asians have long made use of this plant in their local cookery and traditional medicine. The leaves and roots have been well accepted in India as a medicine to treat diabetes mellitus. Their hypoglycemic effects have been demonstrated in both diabetic and normal subjects [9]. The molecular mechanisms responsible for the blood glucose lowering activity of this plant, however, remain unestablished. Ivy gourd has been classified as one of the medicinal herbs in the traditional practices of the ancient Thai medicine with some properties similar to those documented in India [10]. In addition to its anti-diabetic property, the root part has been claimed to have an ability to reduce weight. Evidence of weight loss in overweight patients after administration of the plant extract has been reported in India [9]. Such claims, however, are primarily based on local wisdom and no previous attempts have been made to study the anti-obesity property of ivy gourd in detail. Singh and co-workers [11] have demonstrated that ivy gourd contains an anti-hyperlipidemic element but any profound effects of this plant on adipocyte or adipose tissue functions have not yet been examined. In this study, we have assessed the anti-adipogenic activity of different ivy gourd parts on 3T3-L1 cells. These fibroblastic pre-adipocytes can undergo differentiation in culture and exhibit similar morphology and biochemical properties to *in vivo* adipocytes. They were derived from a cloned subline of Swiss 3T3 mouse embryo fibroblasts and have been widely used to study the adipogenic process *in vitro*[12].

## Results and discussion

### The root extract suppresses intracellular lipid accumulation in induced 3T3-L1 adipocytes during the early stage of adipogenesis

**Figure 1:**
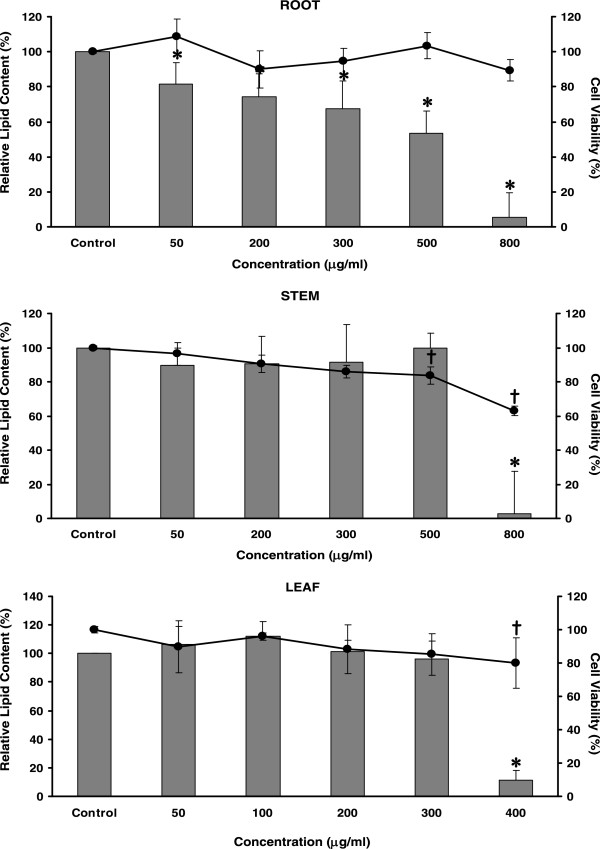
**Effects of an ethanol extract from different parts of ivy gourd on intracellular lipid accumulation and cell viability.** Results are given as a mean value ± S.D. of six-replicate measurements. Bar graphs represent the relative intracellular lipid contents. Asterisks indicate a significant difference at *p* <0.05 from the untreated control (0 μg/mL). Percentages of cell viability are shown as a line graph. Crosses indicate a significant difference at *p* <0.05 from the untreated control (0 μg/mL). The standard TNFα at 10 ng/mL gave 5.00 ± 1.74% of relative lipid content in these experiments.

When we examined the ethanol extracts from the root, stem, and leaf parts of ivy gourd for their anti-adipogenic effects, the root extract apparently lowered the lipid levels in 3T3-L1 adipocytes. The amounts of accumulated lipid in the 3T3-L1 adipocytes following the root extract treatment, measured in terms of the absorbance of the oil red O dye extracted from stained cells, were significantly and dose-dependently decreased (Figure 1). There was a smaller number of fat droplets within the mature adipocytes in the presence of the root extract compared to the untreated cells as revealed by microscopy (Additional file 1: Figure S1). Such an inhibitory effect, did not result from cell damage since toxicity of the root extract was not observed with any of the concentrations tested (Figure 1).

**Figure 2:**
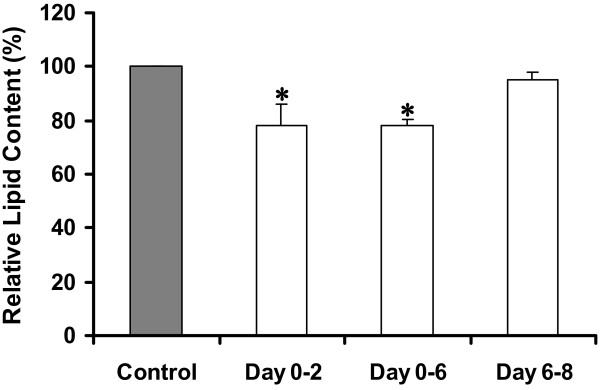
**Effects of the root extract at different treatment times on the accumulation of intracellular lipid.** The relative amounts of accumulated lipid are shown as a mean value ± S.D. of five-replicate measurements for each period that the extract (100 μg/mL) was present in the culture medium. Asterisks indicate a significant difference at *p* <0.05 from the untreated control (0 μg/mL) at Day 8.

The anti-adipogenic effect of the root extract was time sensitive. When we treated 3T3-L1 pre-adipocytes with the extract during differentiation-induction period (Day 0-Day 2) and throughout the course of differentiation (Day 0-Day 6), their intracellular lipid levels decreased equally, regardless of different treatment time-lengths (Figure 2). Administration of the root extract after that period (Day 6-Day 8), however, had no effect on lipid accumulation in the fully differentiated cells (Figure 2). These results indicated that the extract was effective only if introduced early in the adipocyte differentiation program. It acted strictly on differentiating 3T3-L1 pre-adipocytes within the first 2 days of induction with insulin, IBMX (3-isobutyl-1-methylxanthine), and DEX (dexamethasone). A series of adipogenesis-promoting molecules are known to be activated in response to signaling by hormonal inducers [13]. The active compound(s) in the extract might inhibit any of them in some way, thereby blocking adipocyte differentiation at this early stage. Decreasing levels of such critical molecule(s) within the cells during the progress of differentiation were also implicated because the root extract when applied to mature adipocytes (Day 6-Day 8) did not cause any inhibition (Figure 2).Extracts of stems and leaves had no effect on the differentiation of the 3T3-L1 adipocytes. The marked decrease of the lipid contents seen at 400 μg/mL of leaf extract and at 800 μg/mL of treatments with stem extract were really a consequence of their ability to damage the cells based on the MTT assay results (Figure 1). In addition, when we examined the three ivy gourd extracts for their adipolytic activities, none of them exerted lipid-degrading effect on fully differentiated 3T3-L1 adipocytes, as determined from the amounts of glycerol released into the culture medium (data not shown). From the above findings, we then decided to focus on investigating the mechanisms by which the ivy gourd root inhibited the adipocyte differentiation process, and to identify the active elements.

### Hexane fraction of the root extract inhibits adipogenesis in 3T3-L1 cells

**Figure 3:**
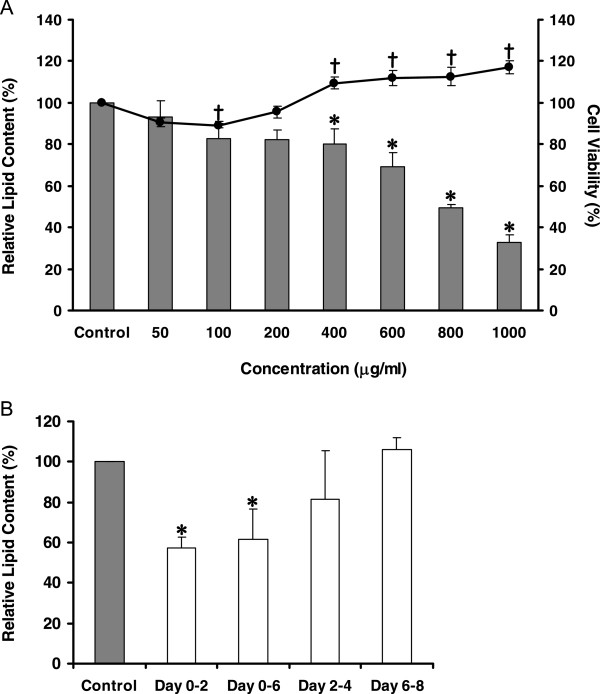
**Effects of hexane fraction from the root extract on the intracellular lipid accumulation and cell viability. (A)** Results are a mean value ± S.D. of six-replicate measurements. Bar graphs represent the relative intracellular lipid contents. Asterisks indicate a significant difference at *p* <0.05 from the untreated control (0 μg/mL). Percentages of cell viability are shown as a line graph. Crosses indicate a significant difference at *p* <0.05 from the untreated control (0 μg/mL). The standard TNFα at 10 ng/mL gave 9.17 ± 3.98% of relative lipid content in these experiments. **(B)** The relative amounts of accumulated lipid are shown as a mean value ± S.D. of five-replicate measurements for each period that the fraction (1,000 μg/mL) was present in the culture medium. Asterisks indicate a significant difference at *p* <0.05 from the untreated control (0 μg/mL) at Day 8.

In an attempt to separate the active components from the root extract by sequential solvent partitioning, five different fractions were obtained (Additional file 2: Figure S3). When we examined each fraction for its ability to inhibit adipogenesis, only the hexane-soluble fraction was active. It could suppress 3T3-L1 adipocyte differentiation during the early stages in a dose-dependent manner without negatively affecting the cell survival (Figure 3), whereas the other fractions were either harmful to the cells or ineffective (Additional file 3: Figure S4). These results indicated that the main constituents in ivy gourd root with a suppressive effect on adipocyte differentiation were likely to be non-polar compounds. The presence of alkaloids [14], fatty acids [15], carotenoids [16], triterpenoids [17], cardenolides [18], a long-chain polyprenol [11], as well as flavonoids, polyphenols, and saponins [19] have been reported in this plant. Although these components have not been shown to inhibit adipogenesis, polyprenol may be one of the potential candidates since it improves dyslipidemia *in vivo*[11]. A current study is underway to identify the active compounds in the hexane fraction.

### PPARγ is the main target for the anti-adipogenic effect of ivy gourd root

Adipogenesis or the process of fat cell formation in 3T3-L1 cells is known to be sequentially regulated by a network of transcription factors and adipogenesis-related genes [13]. When we examined the effects of the root extract on the gene expression of key adipogenesis activators throughout the course of differentiation, there was a significant decrease in both mRNA and protein levels for PPARγ (peroxisome proliferator-activated receptor-γ) and C/EBPα (CCAAT/enhancer binding protein-α) but not C/EBPβ (CCAAT/enhancer binding protein-β) during the early stages as compared with those of the untreated cells (Additional file 4: Figure S2). In general, C/EBPβ induces PPARγ and C/EBPα gene expression [20]. From the above findings, we then assumed that the ivy gourd root extract did not inhibit PPARγ transcription through down-regulation of the expression of C/EBPβ. As a result of PPARγ inhibition, adiponectin and GLUT4 (glucose transporter-4) expressions were decreased (Additional file 4: Figure S2). PPARγ is called the master regulator of adipogenesis. It is important for the promotion and maintenance of the adipocyte phenotype. Typically, PPARγ transcripts and protein levels in the 3T3-L1 cells are elevated within 2 days of the induction period (Day 0-Day 2) and reach their peaks by Day 3-Day 4 [21]. C/EBPα is also known as a major transcription factor of adipogenesis, which functions mainly during the terminal stages of differentiation [20]. PPARγ and C/EBPα coordinately regulate adipocyte-specific gene expression. Their increased levels enhance the mRNA expression of downstream target genes such as FABP4 (aP2), SCD1, leptin, adiponectin (AdipoQ), and GLUT4, leading to the synthesis of several proteins required for intracellular lipid synthesis and storage [22]. GLUT4 and adiponectin are adipogenic markers. They are largely produced in response to the insulin signaling pathway to facilitate cellular uptake of glucose which is ultimately converted into stored lipid. GLUT4 is a transmembrane protein which is necessary for glucose transport into adipocytes [23]. Adiponectin is not only an extensive marker for differentiated adipocytes but also exerts autocrine effects in these cells. This adipokine promotes adipogenesis by stimulating glucose influx through increased GLUT4 gene expression and increased GLUT4 recruitment to the plasma membrane [24]. The suppressed expression of these adipogenesis-promoting genes at both transcriptional and translational levels appears to support our previous findings that the cells treated with the root extract had less intracellular lipid accumulation and fat droplet formation than the untreated controls (Additional file 1: Figure S1).

**Figure 4:**
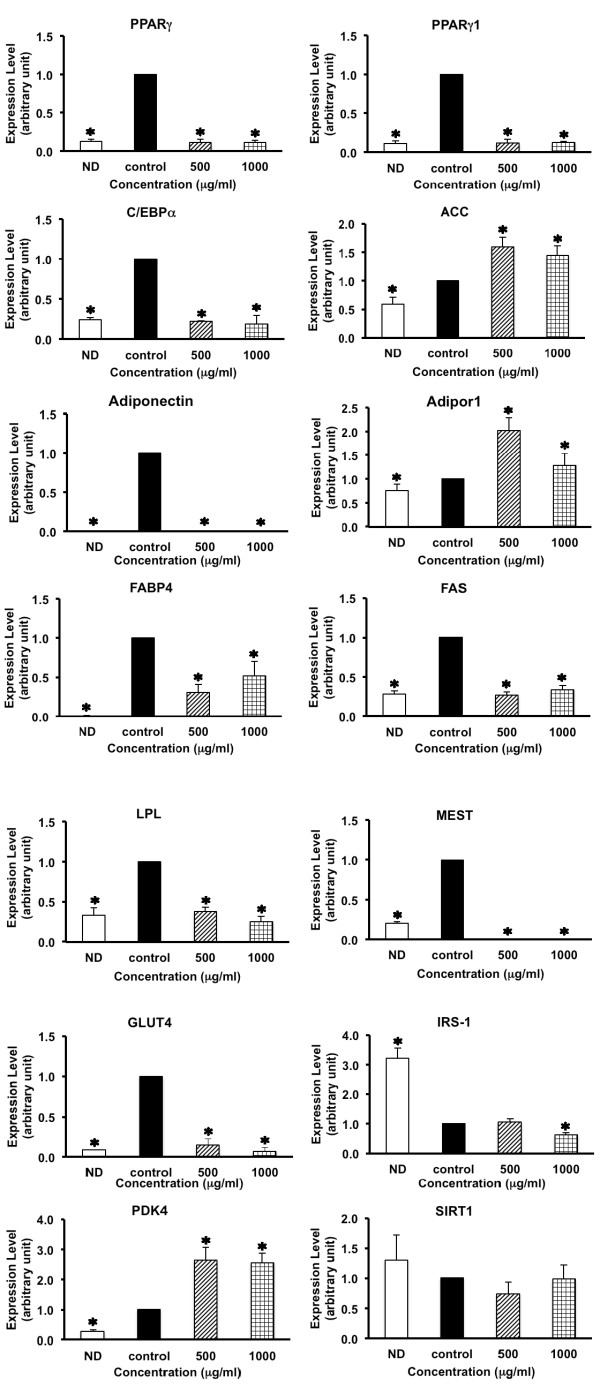
**Effects of hexane fraction on the expression of lipid metabolism-related genes in 3T3-L1 cells.** The cells were cultured in the presence of an ethanol vehicle only (); the hexane fraction at 500 μg/mL (); the hexane fraction at 1,000 μg/mL () during the differentiation induction period (Day 0-Day 2). All cells were harvested at Day 2 to determine the mRNA levels of the interested genes. Their relative expression levels were estimated in terms of their fold-change compared to the β-actin mRNA. Values are shown as a mean value ± S.D. of three-replicateexperimentsfor each treatment. Asterisks indicate a significant difference at *p* <0.05 from the untreated control (0 μg/mL). (ND = non-differentiated 3T3-L1 cells).

In order to address more in detail the molecular mechanisms underlying the suppression of 3T3-L1 cell differentiation by ivy gourd root, we then examined the effects of the hexane fraction on expression of a panel of genes related to lipogenesis during the early stages of adipocyte differentiation. The results are shown in Figure 4. All of the lipogenic genes determined in this study became over-expressed by the hormonal induction of adipogenesis. In accordance with the root extract treatment (Additional file 4: Figure S2), the hexane fraction potently suppressed the up-regulation of PPARγ gene expression in the differentiated cells harvested at Day 2. C/EBPα expression was also decreased after the same treatment. Along with the suppression of PPARγ and C/EBPα, mRNA levels of various adipogenic genes were reduced in the treated cells. FABP4 (fatty acid binding protein-4) is a carrier of fatty acids that plays a supporting role in differentiation of the adipocytes [25]. The down-regulated expression of this gene by the hexane fraction indicated a decline in the ability of the cells to process and metabolize fatty acids, thereby attenuating the intracellular lipid synthesis. Previously, the root extract did not produce any effect on FABP4 expression in the adipocytes (Additional file 4: Figure S2). These conflicting results need to be clarified. It is probable that the extract may contain some chemical components which could counteract such negative effects on the FABP4 gene.

Adipor1 (adiponectin receptor-1) serves as a cell surface receptor for adiponectin. This protein mediates glucose uptake through its binding with adiponectin, thereby promoting lipid synthesis in adipocytes [26]. A large increase in expression of the Adipor1 gene was observed after the hexane fraction treatment, although the mRNA level of adiponectin then substantially decreased. It may be postulated that a decrease in adiponectin output would accelerate the expression of Adipor1 gene in these cells to enhance their adiponectin binding capacities. In the present study, however, we found the alteration only at the transcriptional level. The expression of the protein needs to be examined in order to confirm this hypothesis. The reason for the activated gene expression of ACC1 (acetyl-CoA carboxylase-1) in the treated cells is unknown. The most important function of ACC1 is to provide the malonyl-CoA substrate for biosynthesis of fatty acids [27]. This lipogenic gene is transcriptionally controlled by SREBP1c (sterol regulator element-binding protein-1c) which is also a regulator of the PPARγ gene [28]. The significant down-regulation of MEST (mesoderm specific transcript), an adipocyte size marker gene [29], means that the treated cells became smaller due to their reduced lipid content. In addition, the decreased mRNA level of LPL (lipoprotein lipase) which hydrolyzes triglycerides in lipoprotein particles to provide free fatty acids for intracellular triglycerides synthesis, would further cause reduction in the lipid synthesis and storage of these cells. From these changes in the expression of the lipogenic genes, we then concluded that the hexane fraction suppressed intracellular lipid synthesis in 3T3-L1 adipocytes by negatively modulating both up-stream and down-stream adipogenic genes in the early differentiation pathway.

We also observed some inhibitory effects of the hexane fraction on the glucose metabolism-regulating genes that participate with lipogenesis in the adipocytes. A down-regulation of GLUT4 expression in the treated cells was evident. This observation and those that arose from the extract treatment (Additional file 4: Figure S2) thus indicated that if the ivy gourd root had an anti-diabetic property, it would exert a hypoglycemic effect independently of the glucose transporter-GLUT4 system. PDK4 (pyruvate dehydrogenase kinase-4) transcripts, on the other hand, were significantly increased upon the treatment, and would cause inhibition of the pyruvate dehydrogenase complex [30]. Consequently, conversion of acetyl-CoA from pyruvate was attenuated, and limited its availability for use as a precursor of fatty acids. Surprisingly, SITR1 (sirtuin-1) and IRS1 (insulin receptor substrate-1) which promote cellular glucose metabolism by improving insulin sensitivity [31,32] were not affected by the treatment. The activation of PDK4 expression in combination with the down-regulation of FAS (fatty acid synthase), the key enzyme of fatty acid synthesis pathway, then added negative effects on lipogenesis in the treated differentiating cells.

**Figure 5:**
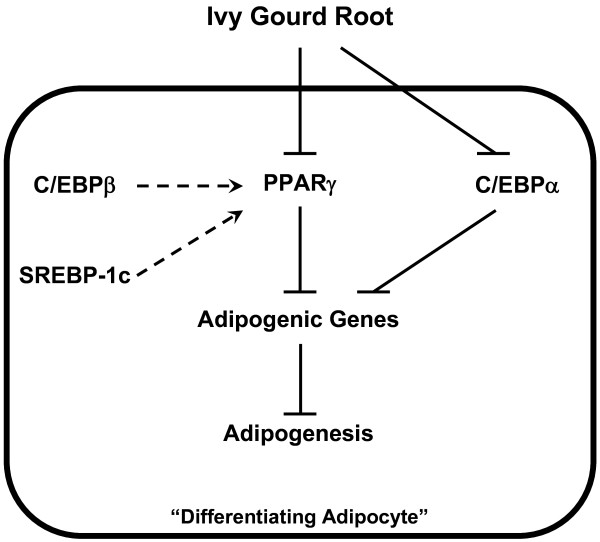
**Proposed molecular mechanisms for the inhibition of adipogenesis by ivy gourd root.** The active constituent(s) in ivy gourd root when introduced to 3T3-L1 cells during the induction of differentiation blocks PPARγ and C/EBPα expressions directly leading to a negative regulation of various adipogenic genes, and eventually inhibits adipogenesis in these cells.

Therefore, the overall anti-adipogenic effect of ivy gourd root in the 3T3-L1 cells seems to be primarily due to down-regulation of the expression of the PPARγ gene early in the differentiation pathway. Our proposed mechanisms as illustrated in Figure 5 include the active element(s) that remain to be identified would initiate the inhibitory effects on the adipocyte differentiation by targeting the PPARγ and also the C/EBPα expression directly, but not through C/EBPβ or SREBP-1c. All the findings from this study imply that the ivy gourd root if applied *in vivo* would prevent or attenuate rather than reduce obesity by mobilizing stored fat from the adipose tissue.

## Conclusion

Our results have shown for the first time, that ivy gourd root possessed an anti-obesity property. It acted directly on pre-adipocytes by inhibiting their differentiation through down-regulation of at least the key adipogenic transcription factor-PPARγ. The presence of possible anti-adipogenic agent in this plant might be relevant to its use to improve metabolic diseases induced by obesity, in addition to having a blood sugar lowering effect. We are now attempting to identify its active component(s). Further study is also necessary to evaluate the anti-obesity effect of ivy gourd root in experimental animals.

## Materials and methods

### Plant materials

Ivy gourd (*Coccinia grandis* L. Voigt) samples were collected in and around Songkhla Province, Thailand. The voucher specimen has been deposited at the Herbarium of the Faculty of Science, Prince of Songkla University.

### Preparation of the extracts

The roots, stems, and leaves were separated, cut into small pieces, and dried at 40°C in a hot-air oven. The dried materials were then ground and macerated with 10 volumes of ethanol. The resulting extracts were then collected, filtered and evaporated to dryness under reduced pressure. The percentage yields of the ethanol extracts from roots, stems, and leaves were 5.74, 8.64, and 12.74 of the initial dry weight, respectively.

### Cell culture and anti-adipogenesis assay

The 3T3-L1 pre-adipocytes were obtained from the American Type Culture Collection (ATCC) and cultured in a humidified atmosphere of 95% air and 5% CO_2_ at 37°C in Dulbecco’s modified Eagle’s medium (DMEM) (GIBCO, USA) containing 10% fetal bovine serum (FBS) (GIBCO, Canada) and penicillin (100 U/mL)-streptomycin (100 μg/mL) (PS) (GIBCO, Canada). Two days after reaching confluence, they were treated with differentiation medium containing 1 μM dexamethasone (DEX; Sigma-Aldrich, USA), 10 μg/mL of insulin (Sigma-Aldrich; USA) and 0.5 mM 3-isobutyl-1-methylxanthine (IBMX; Sigma-Aldrich, USA) in DMEM (designated Day 0). After 2 days for induction of adipogenesis (Day 2), the medium was changed to DMEM containing 10% FBS, PS and 10 μg/mL of insulin for 2 days (Day 4). They were then cultivated in post-differentiation medium (DMEM containing 10% FBS and PS only), which was replaced every 2 days until Day 8. To examine the effects of ivy gourd on adipogenesis, the 3T3-L1 pre-adipocytes were treated with various concentrations of each ivy gourd sample at the time of the induction of differentiation (Day 0-Day 2), a vehicle of dimethyl sulfoxide (DMSO) acted as a negative control and the tumor necrosis factor-α (TNFα) (Sigma-Aldrich, USA) as a positive control [33]. The extent of differentiation was assessed using oil red O staining done on Day 8 and through visual observations under the microscope from Day 1 to Day 8.

### Oil red O staining of the 3T3-L1 adipocytes

The 3T3-L1 adipocytes were washed twice with phosphate buffered saline at pH 7.4 then fixed with 10% formaldehyde for 1 h at room temperature. After the cells were washed with 60% isopropanol, they were stained with oil red O (6 parts of 0.5% oil red O dye in 100% isopropanol to 4 parts of water) for 10 min. After washing with water, three times, the dye stain fixed in the cells was extracted with DMSO and the absorbance (OD) measured at 540 nm. The relative lipid contents were calculated from (OD sample–OD non-differentiated control ÷ OD untreated control–OD non-differentiated control) × 100.

### Cytotoxicity assay

The viability of the cells was measured based on the reduction of yellow MTT [1-(4, 5-dimethylthiazol-2-yl)-3, 5-diphenylformazan] by mitochondrial succinate dehydrogenase to the purple formazan which can only occur in metabolically active cells [34]. In this assay, the 3T3-L1 cells were differentiated in differentiation medium containing each sample for 2 days. They were then cultured in post-differentiation medium supplemented with 0.25 mg/mL of MTT (Sigma-Aldrich, USA) for 2 h at 37°C. The culture medium was removed, and DMSO was added to dissolve the MTT-formazan complex formed. The optical density (OD) was measured at 570 nm. The percentage of cell viability was calculated from (OD sample–OD non-differentiated control ÷ OD untreated control–OD non-differentiated control) × 100.

### RNA analysis

#### Semi-quantitative RT-PCR assay

Total RNA was extracted from the cultured cells using Trizol® reagent (Invitrogen, USA) based on the procedure described elsewhere [35]. First-strand cDNAs were synthesized with M-MLV reverse transcriptase (Bio-Rad, USA) from 2 μg RNA, and were amplified by RT-PCR (MyCycler™ Thermal Cycler System, Bio-Rad, USA) using specific primers and thermal cycling conditions as listed in Additional file 5: Table S1. The expression level of each gene transcript was normalized to the glyceraldehyde-3-phosphate dehydrogenase (GAPDH).

#### Quantitative real-time RT-PCR assay

Total RNA was extracted using the RNeasy mini kit (Qiagen, Germany) according to manufacturer’s instructions. First strand cDNA was generated from 2 μg RNA by the High Capacity RNA-to-cDNA kit (Applied Biosystems, USA). The quantitative real-time RT-PCR (Step One Plus™ Real Time PCR System, Applied Biosystems, USA) was performed at 60°C for 40 cycles for all genes. Their primer sequences are listed in Table 1. The mRNA level of each gene was normalized using β-actin as the internal control.

**Table 1:** Primer sequences for Real-Time PCR

**Gene**	**Forward sequence**	**Accession No.**
**(product)**	**Reverse sequence**	
Actin	5′-CAGAAGGAGATTACTGCTCTGGCT-3′	NM_007393
(93 bp)	5′-GGAGCCACCGATCCACACA-3′	
ACC	5′-GGACCACTGCATGGAATGTTAA-3′	AY451393
(73 bp)	5′-TGAGTGACTGCCGAAACATCTC-3′	
Adiponectin	5′-GTTCCCAATGTACCCATTCGC-3′	NM_009605
(88 bp)	5′-TGTTGCAGTAGAACTTGCCAG-3′	
Adipor1	5′-TCTTCGGGATGTTCTTCCTGG-3′	NM_028320
(104 bp)	5′-TTTGGAAAAAGTCCGAGAGACC-3′	
C/EBPα	5′-TGGACAAGAACAGCAACGAGTAC-3′	AM_007678
(257 bp)	5′-GCAGTTGCCCATGGCCTTGAC-3′	
FABP4	5′-AGCATCATAACCCTAGATGG-3′	NM_024406.2
(115 bp)	5′-CATAACACATTCCACCACCAGC-3′	
FAS	5′- TGCTCCCAGCTGCAGGC -3′	AF_127033
(91 bp)	5′-GCCCGGTAGCTCTGGGTGTA-3′	
GLUT4	5′-CTGCAAAGCGTAGGTACCAA-3′	BC014282
(87 bp)	5′-CCTCCCGCCCTTAGTTG-3′	
IRS1	5′-CCAGAGTCAAGCCTCACACA-3′	NM_010570.4
(179 bp)	5′-GAAGACTGCTGCTGCTGTTG-3′	
LPL	5′-AGGGCTCTGCCTGAGTTGTA-3′	NM_008509
(199 bp)	5′-AGAAATCTCGAAGGCCTGGT-3′	
MEST	5′-GTTTTTCACCTACAAAGGCCTACG-3′	NM_008590
(52 bp)	5′-CACACCGACAGAATCTTGGTAGAA-3′	
PDK4	5′-GAGAAGAGCCCAGAAGACCA-3′	NM_013743
(134 bp)	5′-TCCACTGTGCAGGTGTCTTT-3′	
PPARγ	5′-AGGCCGAGAAGGAGAAGCTGTTG-3′	NM_011146
(276 BP)	5′-TGGCCACCTCTTTGCTGTGCTC-3′	
PPARγ1	5′-AAGATTTGAAAGAAGCGGTGAAC-3′	NM_001127330
(116 bp)	5′-CAATGGCCATGAGGGAGTTAG-3′	
SIRT1	5′-GACGACGAGGGCGAGGAG-3′	NM_019812
(79 bp)	5′-ACAGGAGGTTGTCTCGGTAGC-3′	

ACC, acetyl-CoA carboxylase; Actin, cytoplasmic β-actin; Adipor1, adiponectin receptor-1; C/EBPα, CCAAT/enhancer binding protein transcription factor-α; FABP4, fatty acid binding protein-4; FAS, fatty acid synthase; GLUT4, glucose transporter-4; IRS1, insulin receptor substrate-1; LPL, lipoprotein lipase; MEST, mesoderm specific transcript; PDK4, pyruvate dehydrogenase kinase-4; PPARγ, peroxisome proliferator activated receptor-γ; PPARγ1, peroxisome proliferator activated receptor-γ1; SIRT1, sirtuin (silent mating type information regulation 2 homolog)-1; bp, base pairs.

### Western blot analysis

Briefly, total cell lysate was prepared using a lysis buffer consisting of 0.5 M Tris–HCl, pH 6.8 with 20% glycerol, 20% β-mercaptoethanol, 80 mM dithiothreitol (DTT), and 8% sodium dodecyl sulfate (SDS). Proteins in the lysate were separated by SDS-polyacrylamide gel electrophoresis and transferred onto a polyvinylidene difluoride (PVDF) membrane (Amersham Hybond™-P, GE Healthcare, UK) using an electroblotting apparatus (Mini-PROTEAN® Tetra System, Bio-Rad, USA). The membrane was soaked in 20 mM Tris–HCl, pH 7.6 containing 0.8% NaCl, 0.1% Tween 20, and 5% non-fat dry milk for 1 h at room temperature before further incubated with a primary antibody and a horseradish peroxidase-conjugated secondary antibody for 2 h and 1 h, respectively. After incubation, the membrane was immersed in the chemiluminescent substrate using an ECL assay kit (Super Signal® West Pico, Thermo Scientific, USA). Imaging of blots was then performed on a clear blue x-ray film (CL-XPosure Film, Thermo Scientific, USA) using an automatic x-ray film developing machine (SRX-101A Medical Film Processor, Konica Minolta, Japan) for film processing.

### Statistical analysis

The data are presented as a mean value ± S.D. In each experiment, the inter-group differences were evaluated by one-way ANOVA followed by the Duncan *post hoc* test. Probability values of *p < 0.05* were considered to be significant.

## Abbreviations

ACC: Acetyl-CoA carboxylase; Adipor1: Adiponectin receptor-1; C/EBPα: CCAAT/enhancer binding protein-α; C/EBPβ: CCAAT/enhancer binding protein-β; DEX: Dexamethasone; DIM: Differentiation medium, DMEM, Dulbecco’s modified Eagle’s medium, DMSO, Dimethyl sulfoxide; DTT: Dithiothrietel; ECL: Enhanced chemiluminescence; FABP4: Fatty acid binding protein-4; FAS: Fatty acid synthase; FBS: Fetal bovine serum; GAPDH: Glyceraldehyde-3-phosphate dehydrogenase; GLUT4: Glucose transporter-4; IBMX: 3-Isobutyl-1-methylxanthine; IRS1: Insulin receptor substrate-1; LPL: Lipoprotein lipase; MEST: Mesoderm specific transcript; MTT: 1-(4, 5-dimethylthiazol-2-yl)-3, 5-diphenylformazan; PDK4: Pyruvate dehydrogenase kinase-4; PPARγ: Peroxisome proliferator-activated receptor-γ; PVDF: Polyvinylidene difluoride; RT-PCR: Reverse transcription-polymerase chain reaction; SCD1: Stearoyl-CoA desaturase-1; SDS: Sodium dodecyl sulfate; SITR1: Sirtuin (silent mating type information regulation 2 homolog)-1; SREBP1c: Sterol regulatory element-binding protein-1c; TNFα: Tumor necrosis factor-α.

## Competing interests

The authors declare that they have no competing interests.

## Authors’ contributions

RB performed all the experiments and compiled the data. NHT wrote the manuscript and was responsible for the study concept, designing and coordinating the research, and analyzing the results. KN was responsible for development of the methods. CW was responsible for the experimental designs. MF and HO contributed to designing the study and acquisition of data. All of the authors have read and approved the final form of the manuscript.

## Supplementary Material

Additional file 1: Figure S1 Effects of the root extract on intracellular lipid accumulation. Click here for file

Additional file 2: Figure S3 Fractionation scheme of the ivy gourd root extract. Click here for file

Additional file 3: Figure S4 Effects of four different fractions from the root extract on intracellular lipid accumulation and cell viability. Click here for file

Additional file 4: Figure S2 Effects of the root extract on mRNA and protein expressions of adipogenesis-related genes in 3T3-L1 cells. Click here for file

Additional file 5: Table S1 Primer sequences and conditions for RT-PCR. Click here for file
