# Supplementary figures and images for: Ivy gourd (Coccinia grandis L. Voigt) root suppresses adipocyte differentiation in 3T3-L1 cells
Source: Lipids Health Dis. 2014 May 28;13:88. doi: 10.1186/1476-511X-13-88 (PMC4064515; doi:10.1186/1476-511X-13-88)

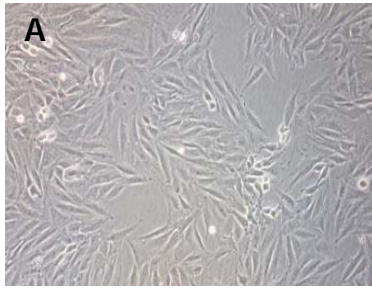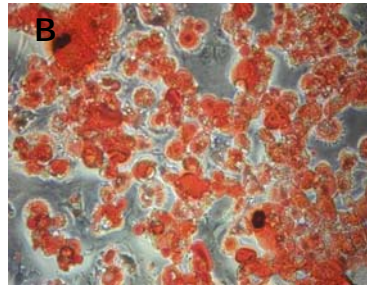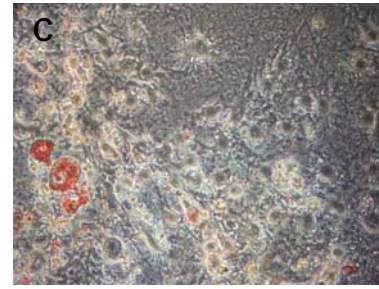

**Figure S1**

Supplement: Additional file 1: Figure S1 — Effects of the root extract on intracellular lipid accumulation. [file 1476-511X-13-88-S1.pdf]

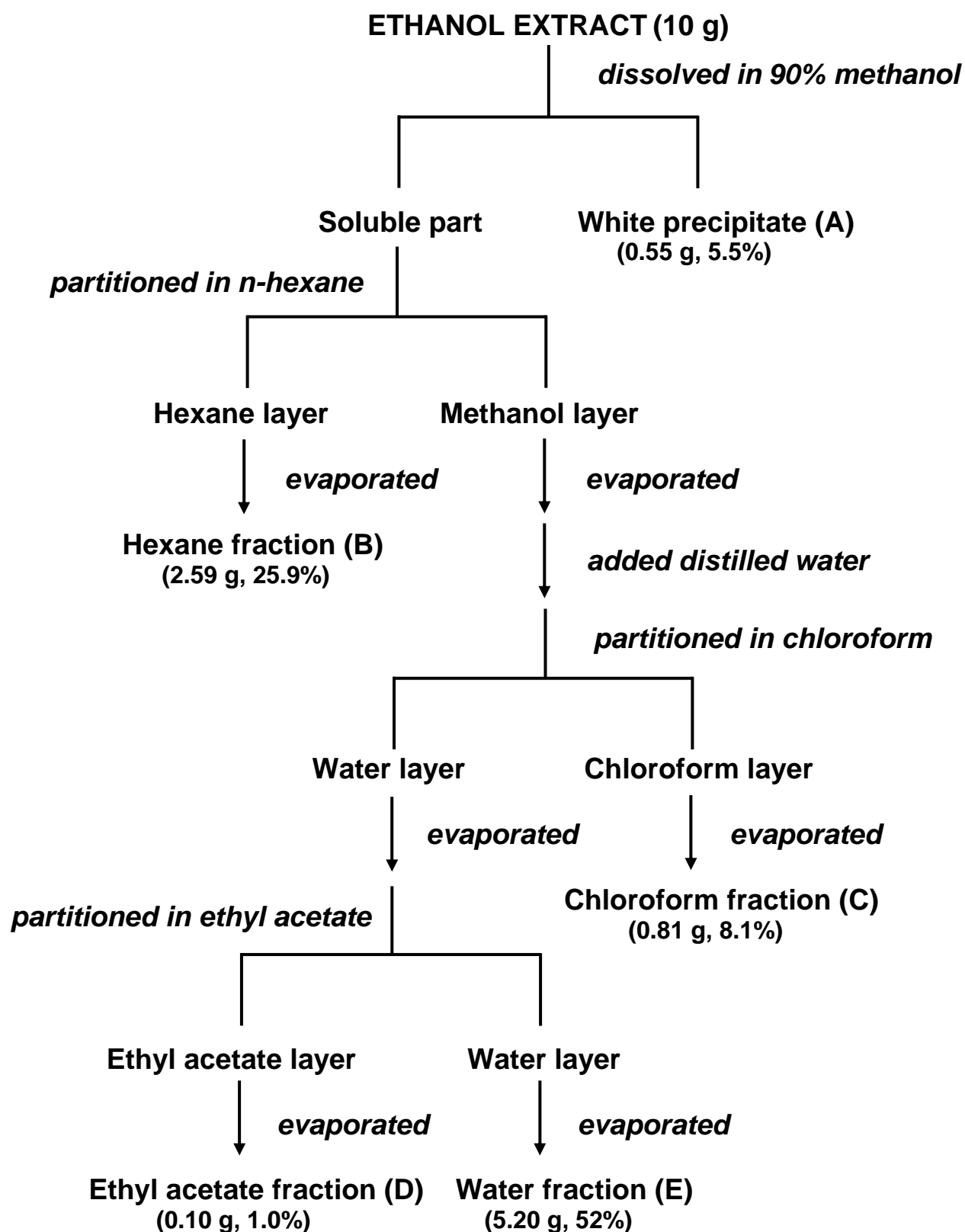

Figure S3

Supplement: Additional file 2: Figure S3 — Fractionation scheme of the ivy gourd root extract. [file 1476-511X-13-88-S2.pdf]

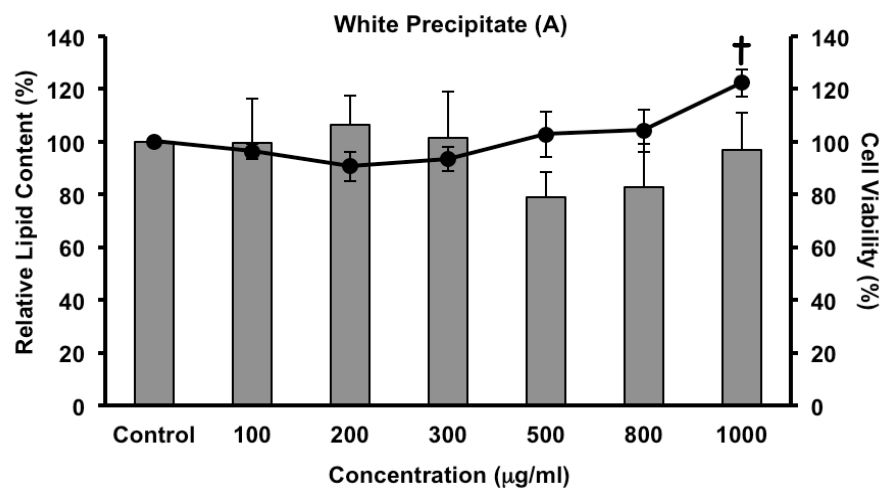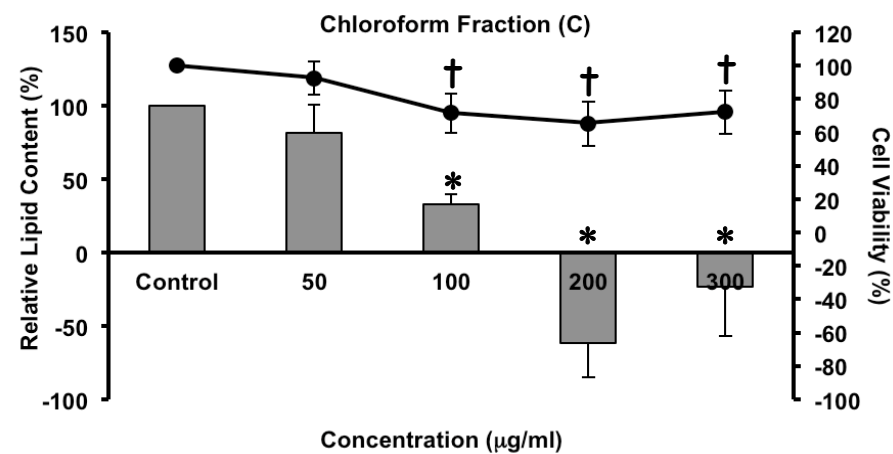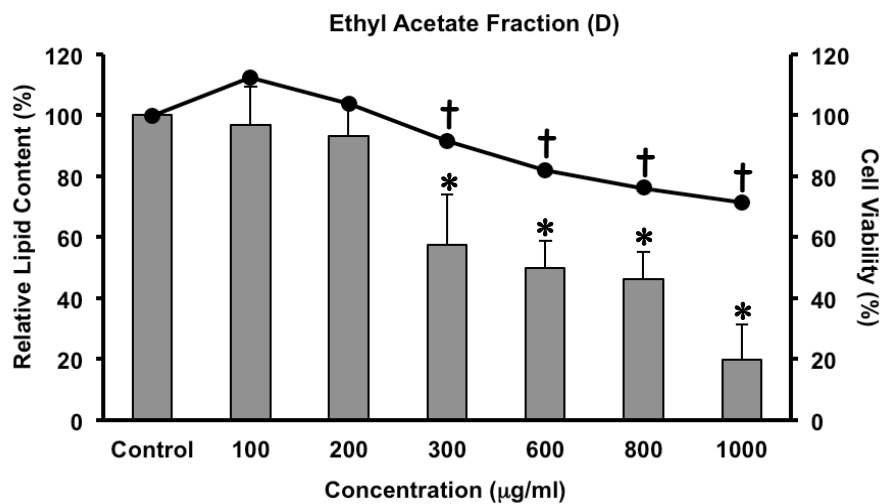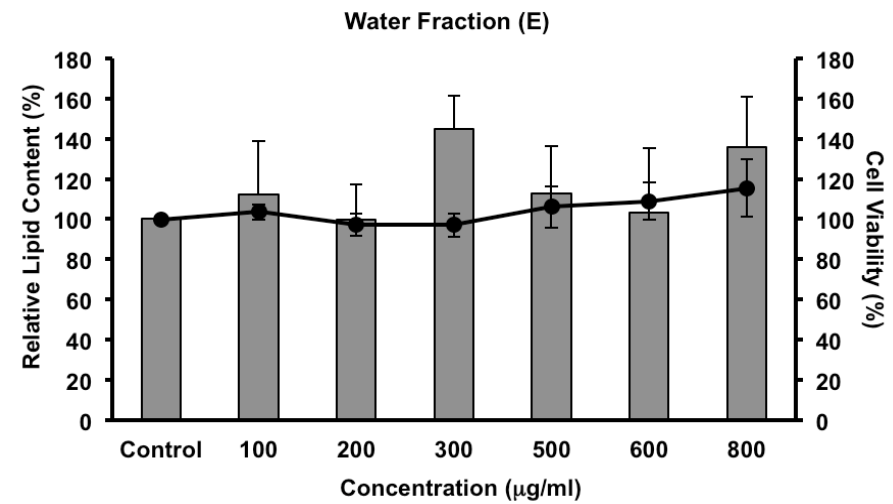

Figure S4

Supplement: Additional file 3: Figure S4 — Effects of four different fractions from the root extract on intracellular lipid accumulation and cell viability. [file 1476-511X-13-88-S3.pdf]

(A)

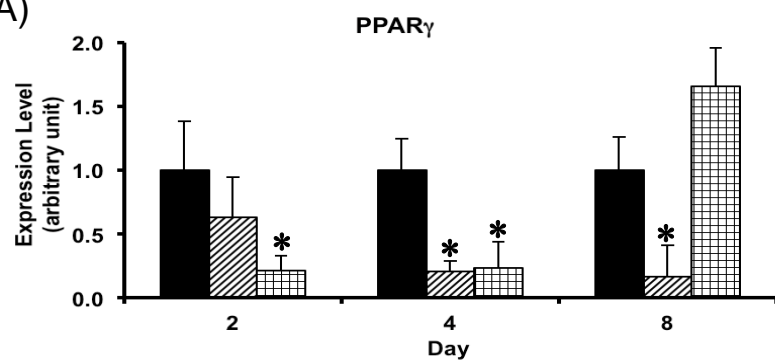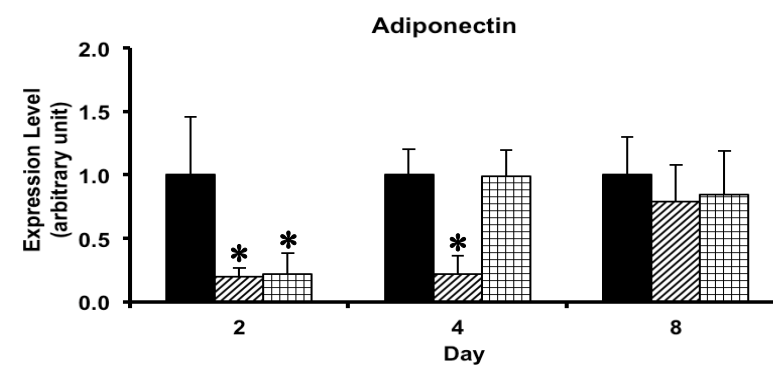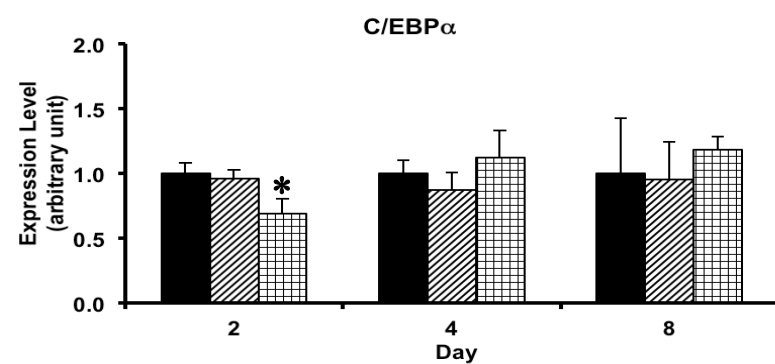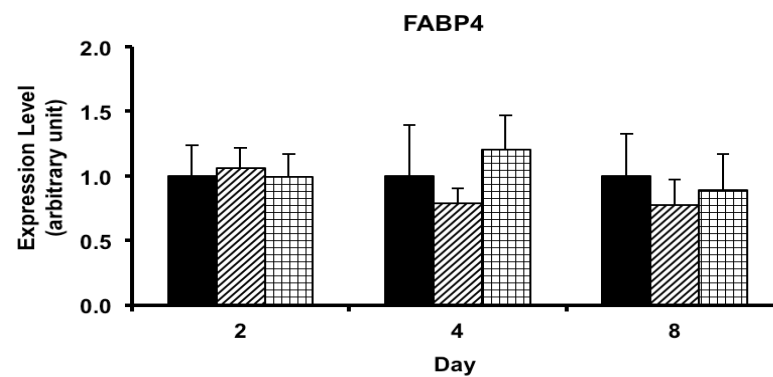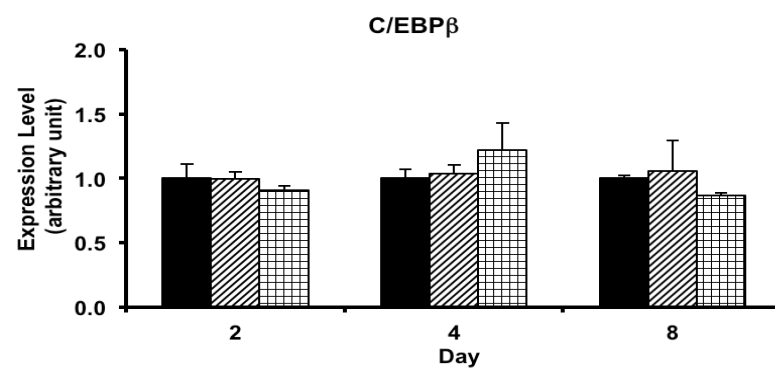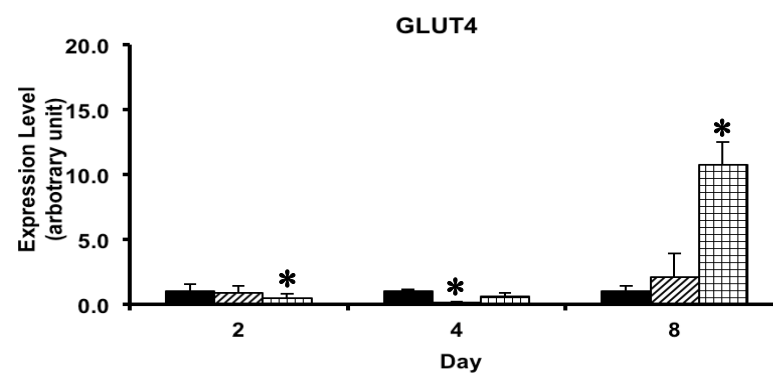

Figure S2

(B)

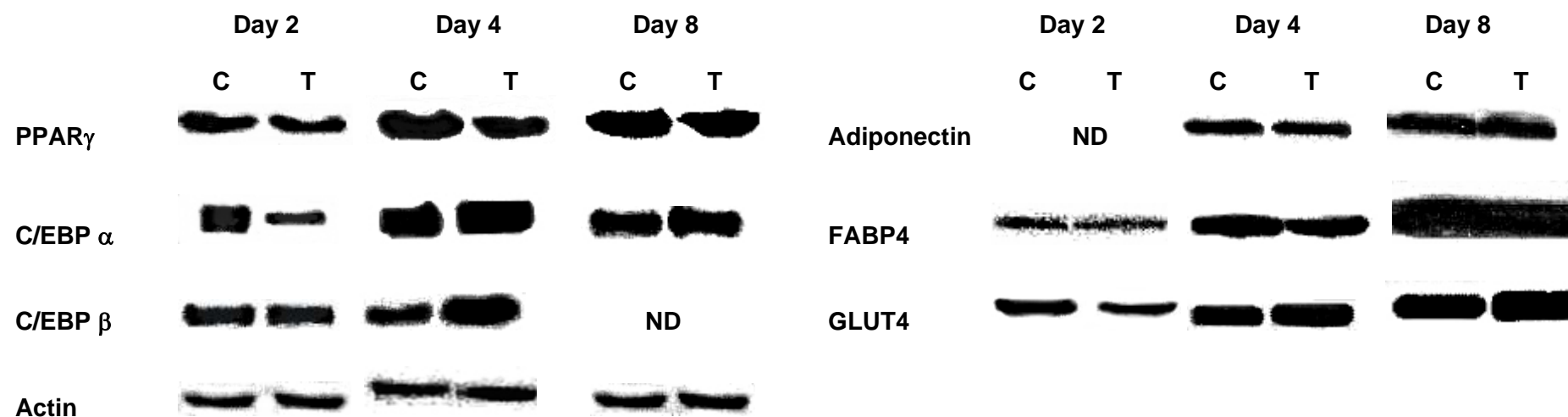

Figure S2 (cont.)

Supplement: Additional file 4: Figure S2 — Effects of the root extract on mRNA and protein expressions of adipogenesis-related genes in 3T3-L1 cells. [file 1476-511X-13-88-S4.pdf]
