# Supplementary material for: Ivy gourd (Coccinia grandis L. Voigt) root suppresses adipocyte differentiation in 3T3-L1 cells
Source: Lipids Health Dis. 2014 May 28;13:88. doi: 10.1186/1476-511X-13-88 (PMC4064515; doi:10.1186/1476-511X-13-88)
Supplement: Additional file 5: Table S1 — Primer sequences and conditions for RT-PCR. [file 1476-511X-13-88-S5.doc]

Table S1 Primer sequences and conditions for RT-PCR

| **Gene Forward sequence PCR Conditions Accession No.**  **(product) Reverse sequence** |
| --- |
| Adiponectin 5’-TGTTGCAAGCTCTCCTGTTCCTCT-3’ 58°C, 35 cycles NM_009605  (552 bp) 5’-AGAGAACGGCCTTGTCCTTCTTGA-3’ |
| C/EBPα 5’-TGGACAAGAACAGCAACGAG-3’ 58°C, 35 cycles NM_007678  (225 bp) 5’-CCTTGACCAAGGAGCTCTCA-3’ |
| C/EBPβ 5’-GGGGTTGTTGATGTTTTTGG-3’ 58°C, 35 cycles NM_009883  (149 bp) 5’-CGAAACGGAAAAGGTTCTCA-3’  FABP4 5’-ACCTGGAAGCTTGTCTCCAGTGAA-3’ 58°C, 35 cycles NM_024406  (291 bp) 5’-TGTGGTCGACTTTCCATCCCACTT-3’ |
| GAPDH 5’-AACTTTGGCATTGTGGAAGGGCTC-3’ 58°C, 35 cycles NM_008084  (382 bp) 5’-TGGAAGAGTGGGAGTTGCTGTTGA-3’  GLUT4 5’-GACGGACACTCCATCTGTTG-3’ 56°C, 35 cycles NM_009204  (400 bp) 5’-GCAGCTGAGATCTGGTCAAAC-3’  PPARγ 5’-GATTCTCCTGTTGACCCAGAG-3’ 62°C, 35 cycles NM_011146  (408 bp) 5’-ACAGACTCGGCACTCAATGG-3’ |

C/EBPα, CCAAT/enhancer binding proteins transcription factor-α; C/EBPβ, CCAAT/enhancer binding proteins transcription factor-β; PPARγ, peroxisome proliferator activated receptor-γ; GLUT4, glucose transporter-4; FABP4, fatty acid binding protein-4; GAPDH, glyceraldehyde-3-phosphate dehydrogenase; bp, base pairs
